# Aesthetic Breast Reconstruction: An Expert Video Roundtable Discussion

**DOI:** 10.1093/asjof/ojae055

**Published:** 2024-07-25

**Authors:** Maurice Nahabedian, Steven Sigalove, Allen Gabriel, Kiya Movassaghi

The authors are pleased to present an expert roundtable discussion on aesthetic breast reconstruction (Video). The discussants include a panel of leading experts in aesthetic surgery, and the video roundtable was recorded live at The Aesthetic Meeting 2024 in Vancouver, Canada. The panelists discussed recent advances in aesthetic breast reconstruction and described how new procedures, including prepectoral reconstruction and nipple sparing mastectomies, have helped improve outcomes and quality of life for patients. They explained the ability of fat grafting to achieve better results and remedy unwanted effects, such as rippling and wrinkling. The panelists concluded by discussing the challenges of aesthetic breast reconstruction, including the loss of sensation that many patients experience, as well as the emerging research, procedures, and technologies that will continue to help aesthetic surgeons minimize these complications and achieve better outcomes for their patients.

